# A computational modeling approach for dosing endoscopic intratumoral chemotherapy for advanced non-small cell lung cancer

**DOI:** 10.1038/s41598-021-03849-w

**Published:** 2022-01-07

**Authors:** Vitor Mori, Jason H. T. Bates, Michael Jantz, Hiren J. Mehta, C. Matthew Kinsey

**Affiliations:** 1 Division of Pulmonary and Critical Care, University of Vermont Medical Center, 89 Beaumont Avenue, Given D208, Burlington, VT 05401 USA; 2 Division of Pulmonary and Critical Care, University of Florida, Gainesville, FL USA

**Keywords:** Lung cancer, Chemotherapy

## Abstract

We recently developed a computational model of cisplatin pharmacodynamics in an endobronchial lung tumor following ultrasound-guided transbronchial needle injection (EBUS-TBNI). The model suggests that it is more efficacious to apportion the cisplatin dose between injections at different sites rather than giving it all in a single central injection, but the model was calibrated only on blood cisplatin data from a single patient. Accordingly, we applied a modified version of our original model in a set of 32 patients undergoing EBUS-TBNI for non-small cell lung cancer (NSCLC). We used the model to predict clinical responses and compared them retrospectively to actual patient outcomes. The model correctly predicted the clinical response in 72% of cases, with 80% accuracy for adenocarcinomas and 62.5% accuracy for squamous-cell lung cancer. We also found a power-law relationship between tumor volume and the minimal dose needed to induce a response, with the power-law exponent depending on the number of injections administered. Our results suggest that current injection strategies may be significantly over- or under-dosing the agent depending on tumor size, and that computational modeling can be a useful planning tool for EBUS-TBNI of cisplatin in lung cancer.

## Introduction

Lung cancer is the leading cause of cancer-related mortality in the world, with 1.76 million deaths in 2018. This is more than the next three deadliest types of cancer combined ^1^. Moreover, even though lung cancer accounts for 13% of new cancer cases, it is responsible for 22% of the deaths^2^. There is a significant interest in therapies that could potentially improve lung cancer response rates.

Endobronchial ultrasound-guided transbronchial needle injection (EBUS-TBNI) of cisplatin has recently emerged as a safe alternative to systemic delivery for treating recurrent centrally located non-small cell lung cancer (NSCLC)^3,4^. The potential advantage of intratumoral delivery of chemotherapy is that it can achieve high concentrations of cytotoxic agent within a tumor while reducing the off-target tissue burden that can lead to adverse side effects ^5,6^. However, there is still no consensus as to exactly how intratumoral cisplatin should be delivered to NSCLC tumors. In fact, only two strategies have been employed clinically to date: (1) administering the entire cisplatin dose as a single central injection^7^, and (2) dividing the dose among five distinct injection sites throughout the tumor^8^. In addition, the dose itself has been chosen empirically, most commonly being up to 40 mg^9^. There would thus seem to be significant opportunity to advance the efficacy of EBUS-TBNI of cisplatin by determining the dose that achieves the best tradeoff between cytotoxicity and systemic side-effects, and by employing an injection strategy that ensures all tumor cells receive a lethal concentration of agent. Indeed, our previous computational modeling work^10^ suggests that there are enormous gains to be made in distributing a given cisplatin dose at optimally chosen injection sites throughout the tumor as opposed to depositing it all at a single location.

The goal of the present work was therefore to investigate how various doses and injection strategies influence treatment response to EBUS-TBNI of cisplatin. As we are not yet at the point where this question can be studied prospectively, we examined the responses achieved in a case series of patients who had received EBUS-TBNI of cisplatin as salvage therapy for late-stage NSCLC. We adapted our previously developed computational model to predict the likelihood of tumor reduction in each patient based on the dose and number of injections they were given, and then compared these predictions to the clinically observed responses. We also used the model to estimate the minimal cisplatin dose required to induce a positive response in each tumor, as well as how this dose depends on the number of spatially distributed injections, with the goal of establishing an initial foundation upon which to set rationalized guidelines for cisplatin dose as a function of tumor volume and delivery strategy.

## Materials and methods

### Patient data

The study was conducted in accordance with the Declaration of Helsinki (as revised in 2013). This study was approved by the University of Vermont (UVM) Committee on Human Research in the Medical Sciences (CHRMS 17-075) and by The University of Florida (UFL) Institutional Review Board (201700864). Informed consent was not required by these oversight committees due to the retrospective nature of the study. A cohort of patients that underwent EBUS-TBNI from January 2009 to November 2018 was retrospectively assessed.

### Computational modeling

**Figure 1:**
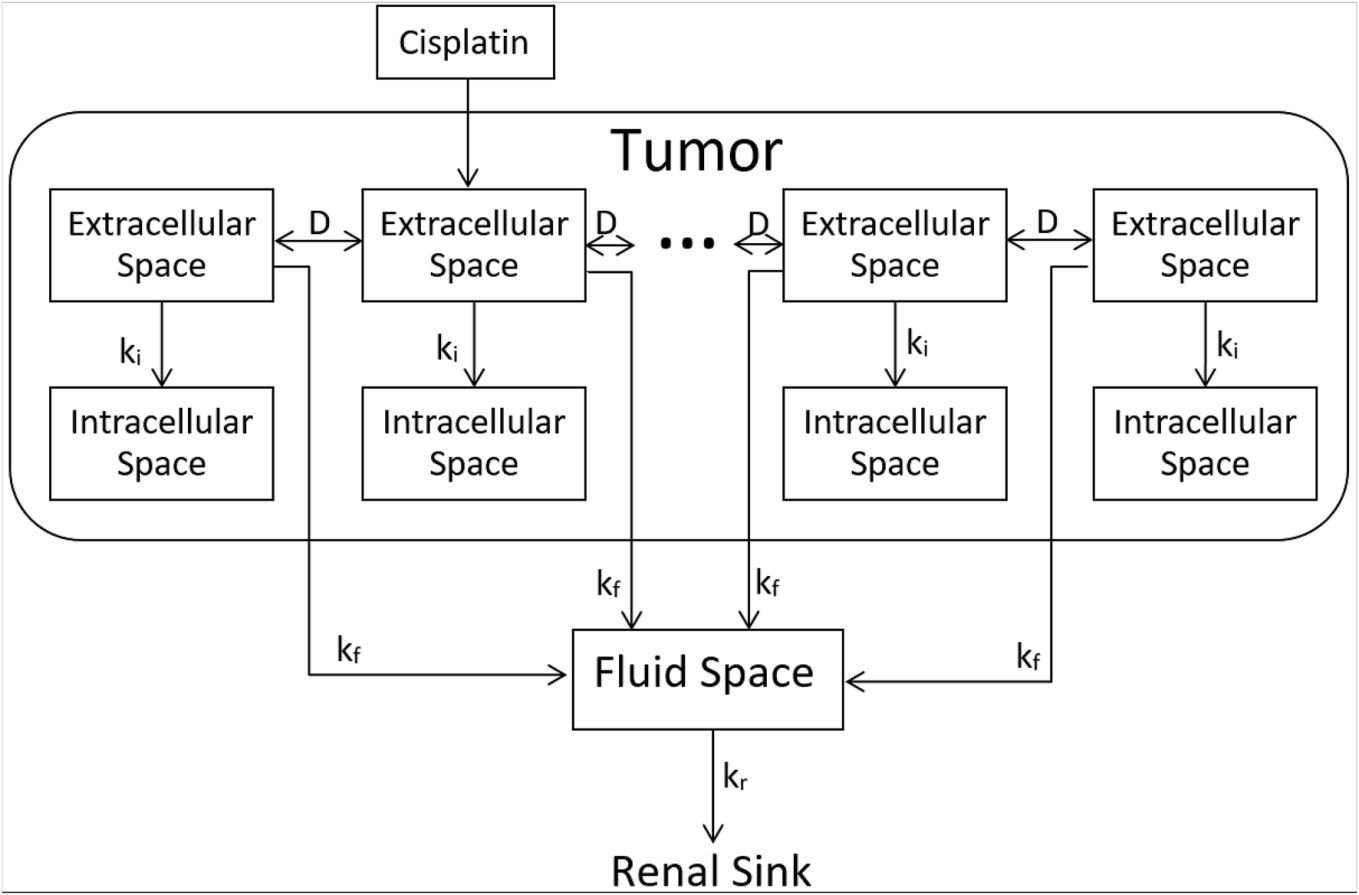
General structure of the computational model of cisplatin pharmacodynamics following intratumoral injections. Each labeled rectangle represents a single well-mixed compartment and arrows represent cisplatin pathways.

We implemented our previously developed computational model of cisplatin pharmacodynamics following intratumoral injection^10^. Briefly, this model considers a tumor to have a boundary defined by segmentation of the tumor in a 3D CT scan^11^. The voxel size reflects the resolution of the 3-D CT scan so that tumor volume and shape can be accurately represented in the model. Volume and shape are the characteristics that distinguish the different tumors from the perspective of our model. Each voxel in the segmented tumor is assumed to be comprised of the superposition of two distinct compartments corresponding to extracellular and intracellular spaces. Cisplatin diffuses within the extracellular compartment $\left({\varphi }_{e}\right)$ with a spatially uniform diffusion constant $D$. It also moves irreversibly into the intracellular compartment $\left({\varphi }_{i}\right)$ with rate-constant ${k}_{i}$, where it exerts its cytotoxic effects. Cisplatin is cleared with rate-constant ${k}_{f}$ by the tumor perfusion and deposited within a systemic fluid compartment $\left({\varphi }_{f}\right)$ having volume ${V}_{f}$ from which it is eliminated via the kidneys with rate-constant ${k}_{r}$. The model is represented diagrammatically in Fig. 1 and mathematically in Eqs. (1–3). ${\varphi }_{e}$, ${\varphi }_{i}$ and ${\varphi }_{f}$ represent the cisplatin concentrations in the extracellular, intracellular and fluid compartments, respectively.

1 $$\frac{d{\varphi }_{e}(\overrightarrow{r},t)}{dt}=D{\nabla }^{2}{\varphi }_{e}\left(\overrightarrow{r},t\right)-\left({k}_{i}+{k}_{f}\right){\varphi }_{e}\left(\overrightarrow{r},t\right)$$

2 $$\frac{d{\varphi }_{f}(t)}{dt}=\frac{1}{{V}_{f}}\frac{d\left[{\int }_{V}{k}_{f}{\varphi }_{e}\left(\overrightarrow{r},t\right)d\overrightarrow{r}\right]}{dt}-{k}_{r}{\varphi }_{f}(t)$$

3 $$\frac{d{\varphi }_{i}(\overrightarrow{r},t)}{dt}={k}_{i}{\varphi }_{e}\left(\overrightarrow{r},t\right)$$

Based on empirical observations^12^, the cisplatin diffusion coefficient within each tissue space was estimated as a function of molecular weight to be $2.47\times {10}^{-6}$ cm^2^/s. The intracellular uptake rate constant was taken from the literature^13,14^ to be $1.05\times {10}^{-4}$/s. The total volume of the fluid space $\left({V}_{f}\right)$ and the cisplatin clearance rate from the tumor to the fluid space $\left({k}_{f}\right)$ were set to be $12.2$ L and $1.46\times {10}^{-4}$/s, respectively. These values are based on the fit of the model to the cisplatin concentrations in the blood from a single patient monitored for 2 h following intratumoral injection^10^.

The model is initialized by loading the initial dose of cisplatin into the extracellular compartment $\left({m}_{j}\right)$, apportioned between 2 and 5 locations $\left(N\right)$ spaced according to our previously described algorithm^10^. Briefly, this algorithm identifies those injection sites that will give rise to the most homogeneous cisplatin distribution pattern within the tumor. Because we assume cisplatin to bind irreversibly to DNA in the intracellular compartment, its concentration in that compartment increases monotonically with time toward an asymptote, ${\varphi }_{\infty }$, defined by the solution of Eqs. (1–3)^10^:

4 $${\varphi }_{i}(\overrightarrow{r},t\to \infty )=\sum_{j=1}^{N}\frac{{k}_{i}{m}_{j}}{D\left|\overrightarrow{r}-{\overrightarrow{r}}_{j}\right|}\cdot {e}^{-\sqrt{\left({k}_{i}+{k}_{f}\right)\cdot \frac{{\left|\overrightarrow{r}-{\overrightarrow{r}}_{j}\right|}^{2}}{D}}}$$

Even though several different features play a role in determining tumor response to treatment, for the sake of simplicity we assume that apoptosis of cancer cells occurs when the intracellular cisplatin concentration exceeds a threshold value, ${\varphi }_{t}$ that was empirically set to be $0.5\times {10}^{-7}$ mg/ml in the original version of the model. In the updated version of the model used in the present study, ${\varphi }_{t}$ is set using the IC_50_ values estimated in vitro from experiments in immortalized human cancer cell lines (Genomics of Drug Sensitivity in Cancer)^15^. We assume that treatment response is related to a 30% reduction in tumor diameter, following RECIST v.1.1 criteria^16^. This is roughly equivalent to a 66% volume reduction for a spherical tumor. Therefore, treatment response will be achieved in the model if at least 66% of the tumor volume reaches an intracellular concentration above ${\varphi }_{t}$. We used the computational model to determine whether or not this was the case for each tumor using the injection strategy that was employed in the tumor in question.

## Results

We screened 37 patients treated for lung cancer with EBUS-TBNI of cisplatin, the inclusion criteria being^7^ age 18–80 years, pathologically confirmed non-small cell lung cancer, histologic or cytologic recurrence of cancer following therapy at initial diagnosis, recurrence in an EBUS-accessible site, and computed tomography (CT) scans performed less than five weeks prior to treatment. The characteristics of the cohort are shown in Table 1.

**Table 1:** Characteristics of the patient cohort.

Characteristics	n (%)
Age^a^	61.8 (8.5)
**Gender**	
Female	20 (54)
Male	17 (46)
Smoking, pack-years^a^	48.7 (25.0)
**Histopathology**	
Adenocarcinoma	15 (40)
Squamous cell	17 (46)
Small cell	4 (11)
Large cell	1 (3)
**Stage**	
I	1 (3)
II	1 (3)
IIIA	16 (43)
IIIB	8 (21)
IV	11 (30)
**Therapy at initial diagnosis**	
Chemotherapy/radiation/surgery	3 (8)
Radiation/surgery	1 (3)
Chemotherapy	1 (3)
Radiation	3 (8)
Chemotherapy/radiation	29 (78)
**Therapy following EBUS-TBNI**	
Chemotherapy	6 (16)
Re-irradiation	3 (8)
Immunotherapy	2 (6)
None	26 (70)

^a^Mean (standard deviation). EBUS-TBNI cisplatin, endobronchial ultrasound-guided transbronchial needle injection of cisplatin.

Out of the 37 patients in the dataset, 33 met the inclusion criteria. We excluded one additional patient with squamous cell lung cancer who passed away prior to assessment of therapeutic response. Of the remaining 32 patients, 15 had confirmed adenocarcinoma, 16 had confirmed squamous-cell carcinoma, and 1 had large-cell carcinoma.

All patients received a total cisplatin dose, determined empirically by the treating physician, ranging from 10 to 40 mg per tumor. Treated lesion response to therapy was determined based on RECIST v1.1 critera^16^. Partial or complete responses by RECIST were considered positive, so the patients were divided into a group that responded to treatment (complete or partial remission) and a group that did not respond (stable or progressive disease). Of the 22 patients treated at the University of Florida, 1 received 2 injections and 21 received 4 injections according to the injected dose, which was 20 or 40 mg, respectively. Of the 10 patients treated at the University of Vermont, all were administered 5 injections regardless of the dose that ranged from 10 to 40 mg. Multiple injections were administered at sites distributed roughly uniformly throughout the tumor as determined empirically by the treating physician, and dose was selected without formal regard to tumor size.

All CT scans were evaluated by an observer who was blinded to the tumor response. Semi-automated segmentation of the treated volume was performed as previously described using the freely available Chest Imaging Platform (https://chestimagingplatform.org)^17^.

Treatment response was observed in 25 patients (78%), with an 80% response rate in patients with adenocarcinoma, a 68.7% response rate in those with squamous-cell carcinoma, and a 100% response rate in patients with large-cell carcinoma.

**Figure 2:**
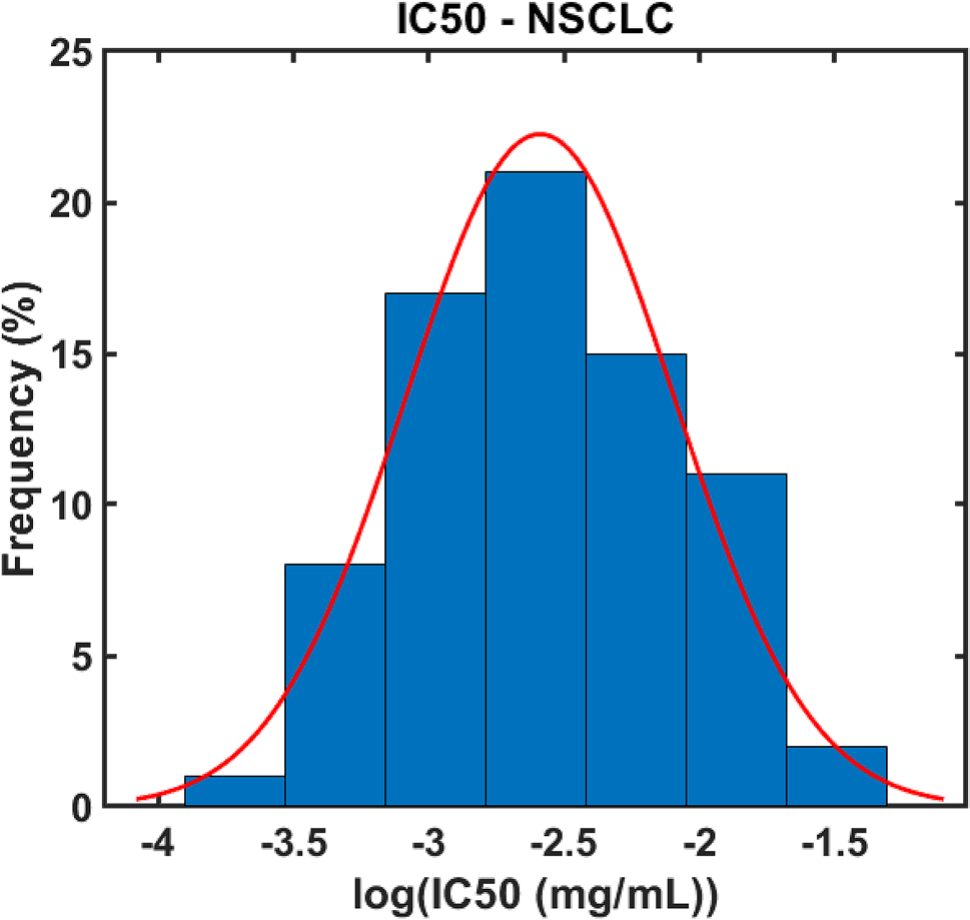
Histogram of the logarithm of 72 h cisplatin IC50 in NSCLC immortalized human cell lines from the Genomics of Drug Sensitivity in Cancer^15^ (blue bars) and fitted normal distribution (red curve).

The dataset from the Genomics of Drug Sensitivity in Cancer platform^15^ showed that the 72-h IC_50_ for cisplatin in 75 immortalized human NSCLC cell lines (adenocarcinoma and squamous-cell) ranged from 0.66 to 155.1 μM ($1.99\times {10}^{-4} \,\mathrm{to} \, 4.65\times {10}^{-2}$ mg/ml). The logarithm of the IC_50_ follows a normal distribution (p > 0.05, Shapiro–Wilk test). Figure 2 shows the distribution of the log-transformed IC_50_ values and the fitted Gaussian curve $\left(\mu =-2.59, \sigma =0.50\right)$. In order to embrace the heterogeneity observed in the IC_50_ values for different cell lines, we drew the threshold intracellular concentration for cell apoptosis in each voxel $\left({\varphi }_{t}\right)$ randomly from the Gaussian distribution shown in Fig. 2.

**Figure 3:**
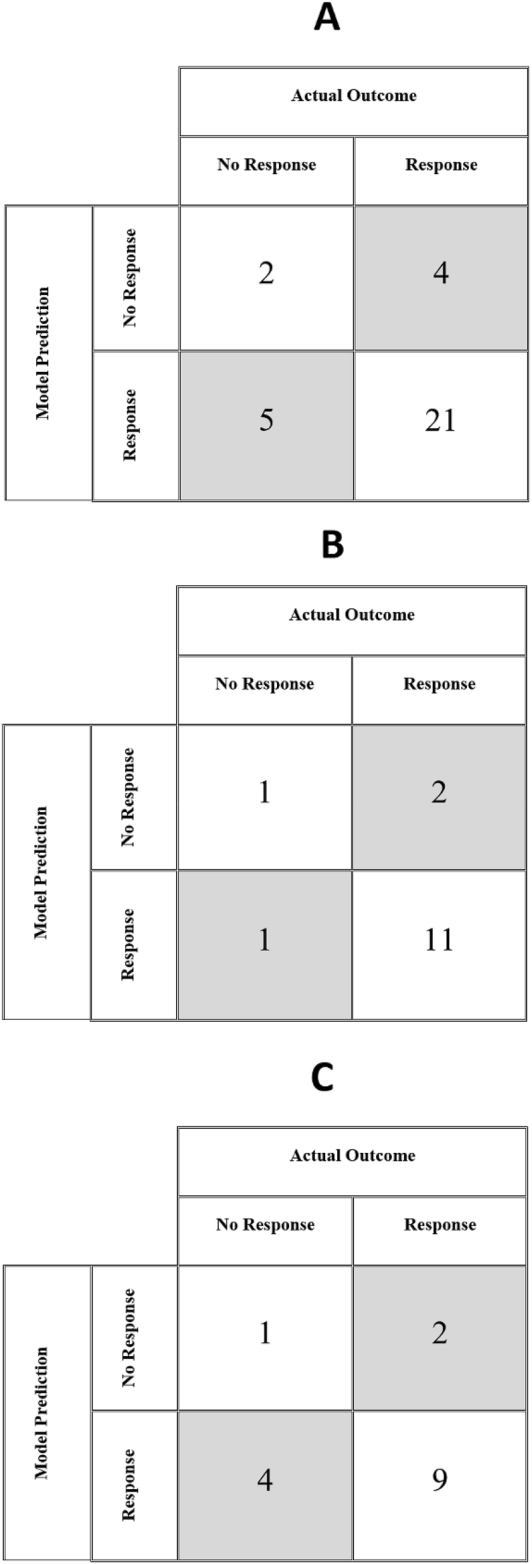
Model results for (**A**) the complete dataset (**B**) adenocarcinoma (**C**) squamous-cell lung cancer.

The model correctly predicted treatment response in 72% of the 32 patients in the dataset. Among the adenocarcinomas, the model performed correctly in 80% of the cases (Table 2). Among the squamous-cell carcinomas, the model performed correctly in 62.5% of the cases (Table 3). The model also correctly classified the outcome for the single case of large-cell carcinoma (Table 4). The confusion matrices for all patients, those with adenocarcinoma, and those with squamous-cell carcinoma are given in Fig. 3A–C, respectively.

**Table 2:** Characteristics of adenocarcinoma patients.

Patient	Cisplatin dose (mg)	Number of injections	Initial tumor volume $\left({\mathrm{cm}}^{3}\right)$	${V}_{response}(\%)$	Model prediction	Clinical outcome
ADN-1	40	4	152.63	18	0	1
ADN-2	40	4	0.95	100	1	1
ADN-3	40	4	4.57	100	1	1
ADN-4	40	4	3.35	100	1	1
ADN-5	40	4	38.04	57	0	0
ADN-6	40	4	6.57	99	1	1
ADN-7	40	4	66.30	32	0	0
ADN-8	40	4	9.44	96	1	1
ADN-9	40	4	13.41	77	1	1
ADN-10	40	4	6.52	98	1	1
ADN-11	40	4	3.27	100	1	1
ADN-12	40	5	1.43	100	1	1
ADN-13	10	5	2.10	99	1	1
ADN-14	30	5	27.77	72	1	0
ADN-15	40	5	229.95	14	0	1

**Table 3:** Characteristics of squamous-cell patients.

Patient	Cisplatin dose (mg)	Number of injections	Initial tumor volume $\left({\mathrm{cm}}^{3}\right)$	${V}_{response}(\%)$	Model prediction	Clinical outcome
SQC-1	40	4	59.71	38	0	0
SQC-2	40	4	5.75	85	1	1
SQC-3	40	4	1.85	100	1	0
SQC-4	40	4	38.49	56	0	1
SQC-5	40	4	14.89	91	1	1
SQC-6	20	2	7.29	82	1	1
SQC-7	40	4	27.04	71	1	1
SQC-8	40	4	1.76	100	1	0
SQC-9	40	4	2.60	95	1	0
SQC-10	40	4	1.12	100	1	1
SQC-11	10	5	28.59	58	0	1
SQC-12	10	5	4.74	97	1	1
SQC-13	10	5	2.73	100	1	1
SQC-14	40	5	15.02	95	1	0
SQC-15	10	5	1.67	100	1	1
SQC-16	40	5	31.59	73	1	1

**Table 4:** Characteristics of large-cell patients.

Patient	Cisplatin dose (mg)	Number of injections	Initial tumor volume $\left({\mathrm{cm}}^{3}\right)$	${V}_{response}(\%)$	Model prediction	Clinical outcome
LCC-1	40	4	1.53	100	1	1

Interestingly, the two tumors associated with the false negatives in the adenocarcinoma group (ADN-1 and ADN-15) were substantially larger (152.6 and 229.9 cm^3^, respectively) than the group mean of 14.1 cm^3^ (SD 19.2 cm^3^) (p = 0.019, Mann–Whitney U test). Among the six small squamous-cell tumors, which all had volumes lower than 4 cm^3^, there were three false positives and three true positives. In the group with tumor volumes > 4 cm^3^ there were two false negatives and one false positive.

We performed a model sensitivity analysis by reassessing its prediction accuracy following an order of magnitude variation in each of the four parameters in turn. These parameters are (1) the diffusion coefficient $\left(D\right)$, (2) the flux rate to the intracellular space $\left({k}_{i}\right)$, (3) the clearance rate to the fluid space $\left({k}_{f}\right)$ and (4) the mean value of the IC50 distribution $\left(\mu \right)$ that determines the intracellular threshold concentration for cell apoptosis $\left({\varphi }_{t}\right)$.

The results are presented in Table 5 and show that the model predictions of therapeutic outcome were essentially unaffected by large variations in model parameter values, with the threshold concentration for apoptosis in the intracellular space being the most sensitive parameter. Indeed, the only meaningful alteration in model prediction occurred with a tenfold reduction in the mean value of the IC50 distribution, implying that particular attention needs to be given to the determination of this quantity^10^.

**Table 5:** Sensitivity analysis of model parameters.

Parameter	True positive (%)	True negative (%)	False negative (%)	False positive (%)
$0.1\cdot D$	62.5	9.4	12.5	15.6
$10\cdot D$	62.5	9.4	12.5	15.6
$0.1\cdot {k}_{i}$	62.5	9.4	12.5	15.6
$10\cdot {k}_{i}$	62.5	9.4	12.5	15.6
$0.1\cdot {k}_{f}$	62.5	9.4	12.5	15.6
$10\cdot {k}_{f}$	62.5	9.4	12.5	15.6
$0.1\cdot \mu$	68.7	6.3	6.3	18.7
$10\cdot \mu$	62.5	9.4	12.5	15.6

**Figure 4:**
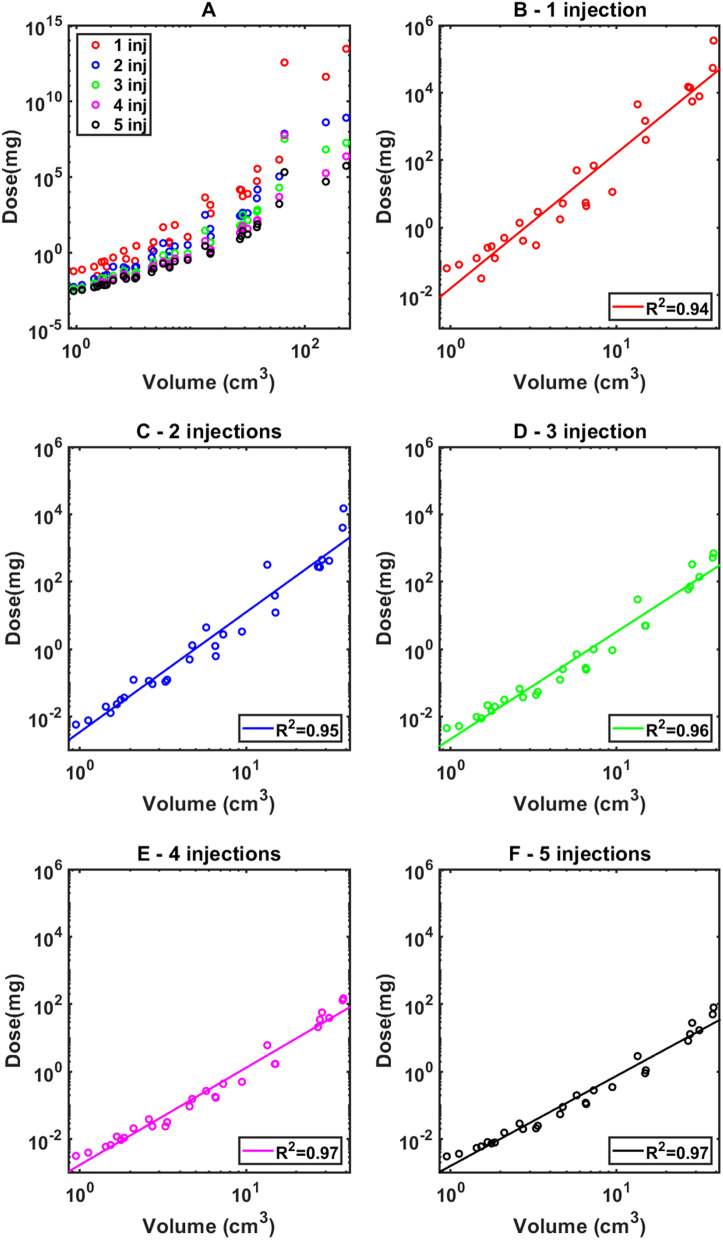
(**A**) Minimum cisplatin dose to achieve a therapeutical response according to the model for 1–5 injections in a log–log scale (**B**–**F**) Minimum cisplatin dose (circles), and power-law regression of ideal dose versus tumor volume for 1–5 injections.

The minimum cisplatin dose estimated by the model to induce a treatment response is non-linearly dependent on tumor volume (Fig. 4A). For tumors < 40 cm^3^ in volume, this relationship is accurately approximated by a power-law of the form $dose=\alpha \cdot {Volume}^{\beta }$, where $\alpha$ and $\beta$ are constants that depend on the number of injections. The estimated values of $\alpha$ and $\beta$ are presented in Table 6. Of note, the exponent of the power-law relationship, $\beta$, decreased systematically as the number of injections increased from 1 to 5 (Fig. 4B–F).

**Table 6:** Parameters estimation for power-law regression of minimum cisplatin dose to induce a response versus tumor volume for 1–5 injections.

Number of injections	$log{}_{10}(\alpha \left(mg\cdot m{l}^{-\beta }\right))$[CI]	$\beta [CI]$	${R}^{2}$
1	$-1.80 [ (-2.17)-(-1.42)]$	$4.00 \left[3.59-4.42\right]$	$0.94$
2	$-2.45 [ (-2.74)-(-2.16)]$	$3.55 \left[3.24-3.87\right]$	$0.95$
3	$-2.66 [ (-2.89)-(-2.43)]$	$3.18 \left[2.92-3.43\right]$	$0.96$
4	$-2.76 [ (-2.94)-(-2.59)]$	$2.88 \left[2.69-3.07\right]$	$0.97$
5	$-2.79 [ (-2.96)-(-2.62)]$	$2.67 \left[2.48-2.85\right]$	$0.97$

## Discussion

Our computational model of cisplatin pharmacodynamics following intratumoral injection in lung cancer is, like all models, based on a set of simplifying assumptions^10^. In the present study, we put these assumptions to the test by comparing the predictions of the model to clinical outcomes in a cohort of non-small cell lung cancer patients receiving EBUS-TBNI of cisplatin (Fig. 3, Tables 2, 3, 4). The predictive accuracy of the model was 62.5% for squamous-cell lung cancers and 80% for adenocarcinoma. Moreover, a sensitivity analysis showed that the model is robust to large changes in the values of its key parameters (Table 5). We believe these findings should be validated, of course, given the limited size of the patient cohort. This reflects the fact that EBUS-TBNI of cisplatin for lung cancer is still an innovative therapy, its sole current indication being as salvage therapy for patients with few other therapeutic options. In fact, the data we used were collected retrospectively from the only two centers in the US that have reported on the use of this therapy, so our cohort remains the largest described. Nevertheless, our findings suggest that the model, despite its many simplifying assumptions, manages to capture important features of cisplatin pharmacodynamics following injection and thus has the potential to serve as a useful adjunct for treatment planning.

Using a literature-based estimate of the IC50 range for cisplatin (Fig. 2), we also estimated the minimum cisplatin dose to achieve a therapeutic response for different delivery strategies as a function of tumor volume (Fig. 4A). Interestingly, we found a strong nonlinear relationship between cisplatin dose and tumor volume that can be well represented by a power-law (Fig. 4B–F) in the 0–40 cm^3^ range, which encompasses 87% of our dataset. This is a potentially important finding that challenges the previously established empirical guideline of 2 mg of cisplatin per ml of tumor^18^. Moreover, a 10–40 mg strategy regardless of volume and number of injections does not seem to capture the myriad of nuances that drive therapeutic response; it is clearly an overdose for small tumors to receive 40 mg in 5 injections, while delivering only 10 mg in a single injection might not be nearly enough for a large tumor.

Due to the nonlinear nature of the power-law relationships identified in Fig. 4, it is apparent that treatment efficacy depends strongly on the number of injections, especially for larger tumors, and that even small variations in the exponent of the power law relationship can result in substantial effects on treatment outcome. For example, for a 40 cm^3^ tumor, the model predicts that 5 injections can reduce the total dose required for efficacy by 3–5 orders of magnitude. On the other hand, dose reduction is only 1–2 orders of magnitude for tumors smaller than 4 cm^3^. This makes intuitive sense given the smaller diffusion distances involved in having cisplatin reach all corners of a smaller tumor, and is also fortuitous given the greater practical challenges of accurately spacing injection sites throughout a smaller tumor. Our model simulations also suggest a formula for calculating cisplatin dosing, up to 40 mg, for a given number of injections according to a power-law relationship with parameters listed in Table 6.

Evaluation of the cases misclassified by the model provides further insight into potential areas for improvement. For adenocarcinomas, two out of the three misclassifications were false negative in tumors significantly larger than the rest of the cohort. Large tumors can develop high interstitial fluid pressures, permanently shutting down small blood vessels that perfuse the tumor. This limits oxygenation in effected areas and causes local necrosis^19,20^. Although necrosis is less common in adenocarcinomas, it can occur in very large tumors^21^. The absence of blood vessels in parts of a tumor limits vascular clearance and so facilitates cisplatin dissemination around the extracellular space, potentially explaining a positive clinical outcome at odds with model predictions for large tumors. Large tumor size may thus suggest the presence of poorly oxygenated areas as a result of necrosis, but this could only be confirmed by histological assessment.

This study has a number of limitations. For example, we did not have the data necessary to calibrate model parameters to the individual patients in our cohort, so the flux of cisplatin to the extratumoral space, and the volume of this space, was based on previous data from a single patient in whom serial plasma concentrations of cisplatin following injection were measured^10^. We used a fixed cisplatin diffusion coefficient estimated from the molecular weight of the drug according to an empirical law for normal tissues at 37 °C^12^. The flux of cisplatin to the intracellular space was based on data from head, neck, and gastric carcinomas^13,14^. Threshold intracellular concentration for cell apoptosis was pulled from immortalized human cell lines (Genomics of Drug Sensitivity in Cancer platform^15^), and this was used to gauge treatment response according to the RECIST v1.1 criteria^16^. Finally, the model does not take into account regional heterogeneities of structure and/or biophysical properties within a tumor, such as may arise spontaneously or as a result of prior chemotherapy or radiation therapy. Nevertheless, the model remained consistent in predicting the therapeutic outcomes of the cohort when parameters were modified by an order of magnitude, demonstrating a degree of robustness in the face of parameter value uncertainties. Going forward, as we develop a greater understanding of how these various factors vary within a tumor and between patients, and develop methods for assessing them in individual tumors, we expect the accuracy of our model predictions to improve accordingly.

The computational model itself also has limitations because of the numerous simplifying assumptions that had to be made in its construction, mostly reflective of a lack of biologic data. Thus, even though we have been able to demonstrate promising performance, we recognize that the ability of the model to predict clinical outcomes could probably be improved substantially with better and more detailed information about the tumor microenvironment. As cytotoxic cisplatin levels within a tumor depend heavily on the competition between drug diffusion and clearance, spatial variations in the cisplatin diffusion coefficient and clearance rate likely impact outcomes significantly. The tumor microenvironment is highly heterogeneous, with densely fibrotic areas^22^ where cisplatin diffusion from sites of injection to distant malignant cells could be particularly limited, yet our model assumes fixed diffusivity throughout the tumor. Relating local diffusivity to Hounsfield unit (HU) values on CT might offer a means of refining the model in this regard, although it remains unclear as to the precise link between diffusivity and HU. Estimating local tissue clearance due to perfusion is even more problematic, as CT does not have the resolution to quantify the microvasculature. In principle, we can estimate global cisplatin clearance from a tumor from the kinetics of cisplatin levels in the blood, as we have shown previously^10^, but unfortunately these data were not available for the patients of the present study. Accordingly, we used an estimated clearance parameter obtained previously in a single patient, but it is likely that this parameter varies significantly between patients, and indeed can even vary over the course of treatment in single patient due to angiogenesis on the time-scale of weeks.

In conclusion, we have demonstrated that our previously developed computational model of cisplatin pharmacodynamics following intratumoral injection has promise as a treatment planning tool for EBUS-TBNI cisplatin of lung tumors, particularly adenocarcinomas. Even in its current form the model suggests that 1–2 injections might be the optimal choice for tumors smaller than 4 cm^3^, while for larger tumors we advocate for as many injections as possible subject to tumor accessibility and patient tolerance. Furthermore, we found a power law relationship between the optimal dose to elicit a clinical response and tumor volume, with the exponent of the power law depending on the number of injections.
